# Structure of the Branched-chain Amino Acid and GTP-sensing Global Regulator, CodY, from Bacillus subtilis*

**DOI:** 10.1074/jbc.M116.754309

**Published:** 2016-12-23

**Authors:** Vladimir M. Levdikov, Elena Blagova, Vicki L. Young, Boris R. Belitsky, Andrey Lebedev, Abraham L. Sonenshein, Anthony J. Wilkinson

**Affiliations:** From the ‡ Structural Biology Laboratory, Department of Chemistry, University of York, York YO10 5DD, United Kingdom,; the § Department of Molecular Biology and Microbiology, Tufts University School of Medicine, Boston, Massachusetts 02111, and; the ¶ STFC Rutherford Appleton Laboratory, Harwell Oxford, Didcot, Oxfordshire OX11 0QX, United Kingdom

**Keywords:** Bacillus, DNA-binding protein, protein structure, transcription repressor, X-ray crystallography

## Abstract

CodY is a branched-chain amino acid (BCAA) and GTP sensor and a global regulator of transcription in low G + C Gram-positive bacteria. It controls the expression of over 100 genes and operons, principally by repressing during growth genes whose products are required for adaptations to nutrient limitation. However, the mechanism by which BCAA binding regulates transcriptional changes is not clear. It is known that CodY consists of a GAF (cGMP-stimulated phosphodiesterases, adenylate cyclases, FhlA) domain that binds BCAAs and a winged helix-turn-helix (wHTH) domain that binds to DNA, but the way in which these domains interact and the structural basis of the BCAA dependence of this interaction are unknown. To gain new insights, we determined the crystal structure of unliganded CodY from *Bacillus subtilis* revealing a 10-turn α-helix linking otherwise discrete GAF and wHTH domains. The structure of CodY in complex with isoleucine revealed a reorganized GAF domain. In both complexes CodY was tetrameric. Size exclusion chromatography with multiangle laser light scattering (SEC-MALLS) experiments showed that CodY is a dimer at concentrations found in bacterial cells. Comparison of structures of dimers of unliganded CodY and CodY-Ile derived from the tetramers showed a splaying of the wHTH domains when Ile was bound; splaying is likely to account for the increased affinity of Ile-bound CodY for DNA. Electrophoretic mobility shift and SEC-MALLS analyses of CodY binding to 19–36-bp operator fragments are consistent with isoleucine-dependent binding of two CodY dimers per duplex. The implications of these observations for effector control of CodY activity are discussed.

## Introduction

In low G + C Gram-positive bacteria, CodY is a global regulatory protein that controls the transcription of numerous genes. In *Bacillus subtilis*, the species in which it was first identified, CodY is a nutrient sensor that represses during growth genes that are turned on under conditions of nutrient limitation (1). The CodY regulon in this organism, encompassing more than 100 genes and operons, encodes degradative enzymes, transporter proteins, catabolic enzymes, antibiotic synthesis pathways, and factors involved in the development of genetic competence and sporulation (2, 3). In human pathogens, such as *Staphylococcus aureus* (4, 5), *Streptococcus pneumoniae* (6), *Streptococcus pyogenes* (7), *Listeria monocytogenes* (8, 9), *Bacillus anthracis* (10), and *Clostridium difficile* (11), CodY also regulates virulence gene expression and provides a regulatory link between metabolism and pathogenesis (12).

CodY from *B. subtilis* and most other low G + C Gram-positive bacteria is a DNA-binding protein that is activated by the branched-chain amino acids (BCAAs),^2^ leucine, isoleucine and valine, which may be viewed as signals of the metabolic status of the cell, and GTP, which may signal the energetic status of the cell (13, 14). During growth in rich media, the concentrations of these species are high, and CodY binds to numerous target promoters and represses transcription of many genes while activating others (2). As the cells begin to experience nutrient limitation, the concentrations of BCAAs and GTP drop, leading to dissociation of CodY from the DNA and derepression of genes that are required for adaptation to nutrient-poor conditions. In the majority of cases, CodY is a repressor competing for DNA binding with RNA polymerase or with other positive regulators or causing premature termination of transcription by a roadblock mechanism (15). At some target genes, CodY acts as an activator either directly or indirectly (2, 16, 17). CodY binds to a 15-nucleotide canonical consensus sequence AATTTTCWGAAAATT that was first identified for the protein from *Lactococcus lactis* (18, 19) and later shown to play an important role in *B. subtilis* (20).

The scope and complexity of CodY regulation of gene expression in *B. subtilis* have been revealed by genome-wide studies (2, 3, 21, 22). These identified the substantial complement of CodY-binding sites on the chromosome of *B. subtilis* and the broad range of affinities CodY has for these sites (22), consistent with the notion that the threshold concentration of CodY activity needed to trigger a transcriptional response varies from gene to gene. This gives rise to hierarchical control of gene expression in the CodY regulon allowing differential transcriptional responses according to the extent of nutrient limitation (3).

CodY from *B. subtilis* consists of 259 amino acid residues. The crystal structures of two fragments of CodY from *B. subtilis* that constitute its effector (residues 1–155) and DNA binding (residues 168–259) domains have been solved (23). CodY(1–155) is a dimer of GAF (cGMP-stimulated phosphodiesterases, adenylate cyclases, FhlA (24)) domains. It consists of three tiers, a basal three-helix bundle that forms a dimerization surface, a central five-stranded β-sheet, and a distal region formed by two extended loops that connect adjacent strands on the sheet. In complexes with isoleucine or valine, these loops embrace the ligand; comparison with the uncomplexed GAF domain reveals up to 15 Å displacements of backbone atoms in these loops upon effector binding (25). The C-terminal fragment CodY(168–259) has a winged helix-turn-helix (wHTH) domain fold.

These structures provided insights into the mode of BCAA binding and a basis for interpreting sequence conservation in CodY orthologues, but they gave no insights into the juxtaposition of the domains in the intact molecule, and thus it was not possible to infer how effector binding alters the structure of the protein so as to regulate DNA binding. Here, we describe the crystal structure of full-length CodY in the unliganded state, together with that of a point mutant, CodY(L3S), in complex with isoleucine. These data, together with light scattering data on CodY and its complexes with DNA in solution, allow a mechanism for isoleucine control of DNA binding by CodY to be proposed.

## Results

### 

#### 

##### Domain Organization in CodY

The crystallization and the preliminary X-ray analysis of the full-length CodY protein were reported previously (26). However, the crystals were not easy to reproduce, and we were unable to solve their structure either by *ab initio* phasing or by molecular replacement using the coordinates for the isolated GAF domain in complex with isoleucine together with the coordinates for the wHTH domain (23). The structure of the unliganded form of the GAF domain (25) provided a search model that allowed successful structure solution by molecular replacement methods.

We have solved the structure of unliganded CodY in three different crystal forms containing 14 (form A), 10 (form B), and 2 (form C) molecules in the asymmetric unit from data sets extending to 4.6, 3.0, and 3.7 Å spacing, respectively (Table 1). These crystals were grown in the presence of different buffers, precipitants and additives as described under “Experimental Procedures.” For two of the crystallizations, GTP or a GTP analogue was present although neither was observed bound to the protein. The following descriptions relate to the structure of the form B crystals, because these crystals diffracted to the highest resolution (3.0 Å). In addition, we have solved the structure of a leucine 3 to serine mutant, CodY(L3S), in complex with isoleucine at 3.0 Å resolution in a crystal form containing four molecules per asymmetric unit (Table 1). We have been unable to obtain crystals of either liganded wild-type CodY or unliganded CodY(L3S) suitable for X-ray analysis.

**TABLE 1:** **X-ray data collection and refinement statistics**

Ligand/molecules per AU	CodY (form A) −/14	CodY (form B) −/10	CodY (form C) −/2	CodY(L3S) Ile/4
**Protein Data Bank ID**	5LOO	5LNH	5LOJ	5LOE

**Data collection**				
X-ray source	SRS beamline PX14.1	SRS beamline PX14.2	ESRF beamline M14	SRS beamline PX10.1
Wavelength (Å)	0.98040	0.97820	0.93300	0.98000
Collection temperature (K)	100	100	100	100
Resolution range (Å)	20.00–4.60	50.00–3.00	25.00–3.70	50.00–3.00
Space group	C121	C121	P4_3_22	P2_1_2_1_2
Unit-cell parameters (Å,°)	*a*= 315.63, *b*= 113.68, *c* = 168.59	*a* = 138.87, *b* = 110.55, *c* = 257.41	*a* = 111.52, *b* = 111.52, *c* = 119.54	*a* = 134.69, *b* = 158.88, *c* = 55.412
	α = 90.0, β = 113.23, γ = 90.0	α = 90.0, β = 91.3, γ = 90.0	α = 90.0, β = 90.0, γ = 90.0	α = 90.0, β = 90.0, γ = 90.0
Matthews coefficient/solvent content (%)	2.9/57.8	3.3/62.9	3.2/60.5	2.5/50.5
No. of unique reflections, overall/outer shell*^a^*	2967/1922	57,358/1376	8235/813	22,830/1151
Completeness (%), overall/outer shell*^a^*	73.6/71.6	72.0/17.4	97.5/98.2	92.5/47.3
*I*/σ(*I*), overall/outer shell*^a^*	6.8/10	18.3/0.51	33.1/5.6	25.3/1.03
*R*_merge_*^b^* (%), overall/outer shell*^a^*	9.0/50.8	10.2/−	5.9/40.8	6.5/65.4

**Refinement and model statistics**				
*R*-factor*^c^* (*R*-free*^d^*)	0.251 (0.429)	0.230 (0.279)	0.233 (0.388)	0.238 (0.290)
Reflections (working/free)	27,647/1479	53,946/2880	7,842/381	21,607/1164
Outer shell *R*-factor*^c^* (*R*-free*^d,e^*)	0.357 (0.423)	0.425 (0.439)	0.258 (0.407)	0.359 (0.389)
Outer shell reflections (working/free)*^e^*	2577/162	837/53	541/33	728/44
Molecules/asymmetric unit	14	10	2	4
No. of protein non-hydrogen atoms	28,546	20,090	4004	8187
R.m.s. deviation from target*^f^*				
Bond lengths (Å)	0.011	0.018	0.019	0.012
Bond angles (°)	1.441	1.607	1.157	1.298
Average *B*-factor (Å^2^)	235.09	86.52	80.43	96.32
Ramachandran plot*^g^*	73.1/17.6/9.4	95.5/3.7/0.8	71.7/17.2/11.1	93.6/4.5/1.9

*^a^* The outer shell corresponds to 4.58–4.50 Å, 3.11–3.00 Å, 3.83–3.70 Å, and 3.11–3.00 Å.

*^b^ R*_merge_ = Σ*_hkl_*Σ*_I_*|*I_I_* − 〈*I*〉|**/**Σ*_hkl_*Σ*_I_*〈*I*〉, where *I_I_* is the intensity of the *i*th measurement of a reflection with indexes *hkl* and 〈*I*〉 are the statistically weighted average reflection intensity.

*^c^ R*-factor = Σ‖*F_o_*| − |*F_o_*‖/Σ|*F_o_*|, where *F_o_* and *F_c_* are the observed and calculated structure factor amplitudes, respectively.

*^d^ R*-free is the *R*-factor calculated with 5% of the reflections chosen at random and omitted from refinement.

*^e^* Outer shell for refinement corresponds to 4.611–4.500 Å, 3.078–3.000 Å, 3.800–3.706 Å, and 3.078–3.000 Å, respectively.

*^f^* Root mean square deviation of bond lengths and bond angles from ideal geometry.

*^g^* Percentage of residues in preferred/allowed/outlier.

**FIGURE 1.**
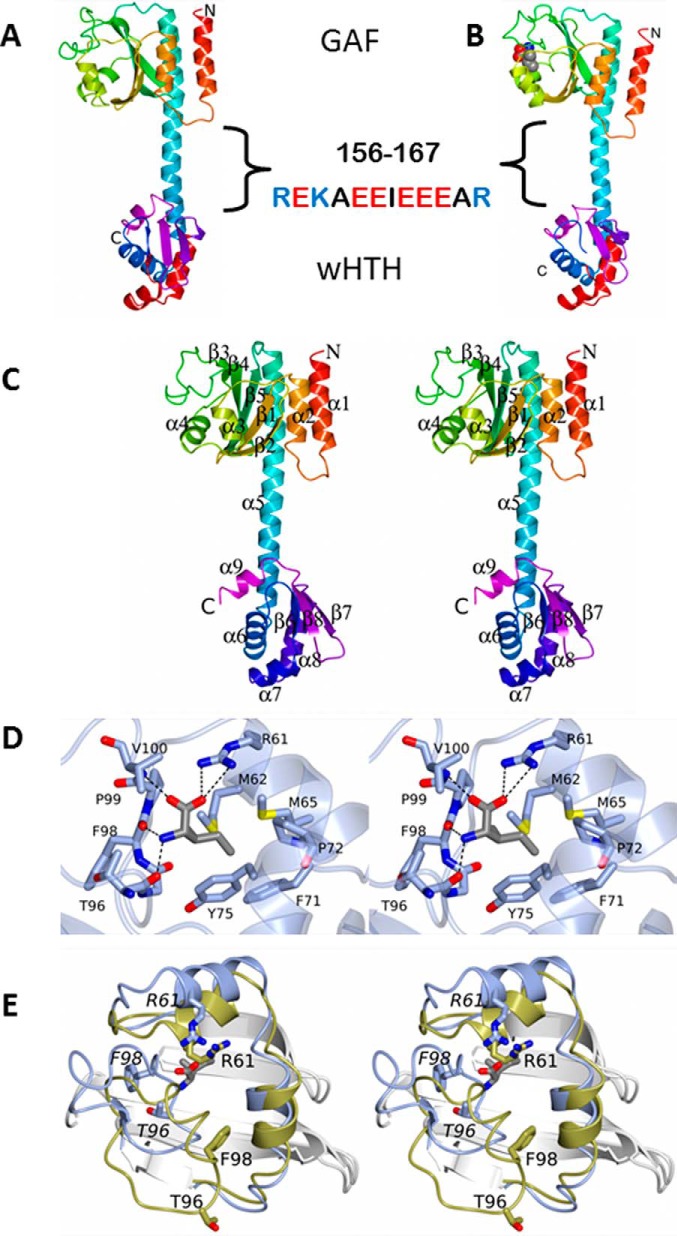
**Domain structure of CodY and the ligand-binding site.** *Ribbon* renderings of single chains from the crystal structures of unliganded CodY (*A*) and the CodY(L3S)-isoleucine complex (*B*) are shown. The chains are color ramped from the N terminus (*red*) to the C terminus (*magenta*) with the helix-turn-helix motif of the wHTH domain also colored in *red.* The isoleucine ligand is shown as van der Waals spheres colored by atom type as follows: carbon, *gray*; oxygen, *red*; nitrogen, *blue*. The GAF and wHTH domains are labeled. The sequence of the glutamate-rich central domain is also highlighted. *C,* stereo representation of the unliganded CodY structure with the secondary structure elements labeled. *D* and *E,* stereo views of the ligand binding pocket. *D*, residues surrounding the isoleucine effector are shown with polar protein-ligand interactions represented by *dashed lines*. The carbon atoms of the protein are shown in *light blue* and sulfur atoms are *yellow. E*, GAF domains of unliganded CodY and the isoleucine-liganded protein are superimposed and shown as *white ribbons* with the loops connecting β2 and β3 and β3 and β4 colored in *gold* and *blue*, respectively. The side chains of Thr-96, Phe-98, and Arg-61 are labeled in *normal font* in the unliganded domain and in *italic font* for the isoleucine complex so as to illustrate the extent of the conformational changes that accompany ligand binding.

The full-length CodY protomer has an elongated structure that spans 80 Å in the longest dimension with a dumb-bell shape (Fig. 1). The 12 residues (156–167) that link the previously reported GAF and wHTH domain structures are in an α-helical conformation. They extend helix α5 in the GAF domain and connect it to what was previously named helix α6 in the wHTH domain, thereby creating a single α-helix spanning residues 137–177. For the full-length CodY, the secondary structure elements are renumbered accordingly in Fig. 1*C*. The inter-domain linker portion of this helix has an unusual sequence with 9 of the 12 residues in the 156–167 segment possessing ionizable side chains, including five glutamates in a string of six residues. Within the CodY chain, there are no non-covalent interactions between the domains that exist as discrete entities. As a result, the isoleucine-binding site in the GAF domain and the helix-turn-helix, which constitutes the DNA-binding site in the wHTH domain, are remote from one another (Fig. 1, *A* and *B*).

##### Branched-chain Amino Acid Binding Pocket

Isoleucine was bound to the GAF domains of all four subunits in the CodY(L3S) tetramer. The ligand was almost completely enclosed in a pocket formed by the β2-β3 and β3-β4 segments of the polypeptide (Fig. 1, *B* and *D*). This pocket is distal to the site of the leucine 3 to serine substitution that resides in helix α1 at the N terminus. The ligand carboxylate forms an ion pair with the side chain of Arg-61 and a further polar contact with the main chain amide of Val-100, whereas its amino group forms polar contacts to the main chain carbonyl groups of Thr-96 and Phe-98 (Fig. 1*D*). The isobutyl side chain of the isoleucine ligand projects into the protein parallel to the β-sheet and is surrounded by the side chains of Met-62, Met-65, Phe-71, Pro-72, Tyr-75, and Pro-99 (Fig. 1, *B* and *D*). Various amino acid substitutions at positions 61, 71, and 98 have been shown to have a range of effects on CodY activity (3, 27–29). This mode of isoleucine binding to CodY(L3S) exactly recapitulates that seen previously in the structure of isolated GAF domain of the native protein (23). When comparing chains from the CodY(1–155) and full-length CodY(L3S) crystal structures, the average pairwise root mean squared deviation (r.m.s.Δ) of equivalent Cα atoms is 0.7 Å. We conclude that ligand binding is essentially unchanged in CodY(L3S) and that the effects of the leucine 3 to serine mutation on the tertiary structure of the GAF domain are barely discernible.

As may be seen in Fig. 1*E*, in the absence of ligand, the GAF domain structure in CodY is significantly altered in the vicinity of the ligand-binding site. The positional r.m.s.Δ following least squares superposition of 154 equivalent GAF domain Cα atoms is 3.4 Å. However, the structural changes are unevenly distributed (133 Cα superpose with an r.m.s.Δ = 1.2 Å) because very large deviations are seen in residues 94–108. Here, >10-Å atomic positional displacements are observed as the β3-β4 segment undergoes dramatic rearrangement accompanying the binding and release of the ligand, so much so that the ligand binding pocket is not formed in the absence of the ligand. Again, the unliganded GAF domain conformation seen in the full-length protein resembles that seen in the crystal structure of unliganded CodY(1–155) with a positional r.m.s.Δ of 0.4 Å for equivalent Cα atoms. As a result, the structural changes accompanying isoleucine binding to the full-length protein closely match those reported previously for ligand binding to the isolated GAF domain (25).

##### Quaternary Structure in the Crystal

Examination of the molecular packing in the crystals of unliganded CodY reveals that the basic building block of all three crystals is a tetramer. The tetramer in the form C crystals and one of the tetramers in both the form A and the form B crystals are generated by crystallographic 2-fold symmetry. Superposition of the eight tetramers from the three crystal forms suggests that they are essentially identical within the limits of the data. As before, the following description relates to a tetramer from the form B crystals because these crystals diffracted to the highest resolution.

**FIGURE 2.**
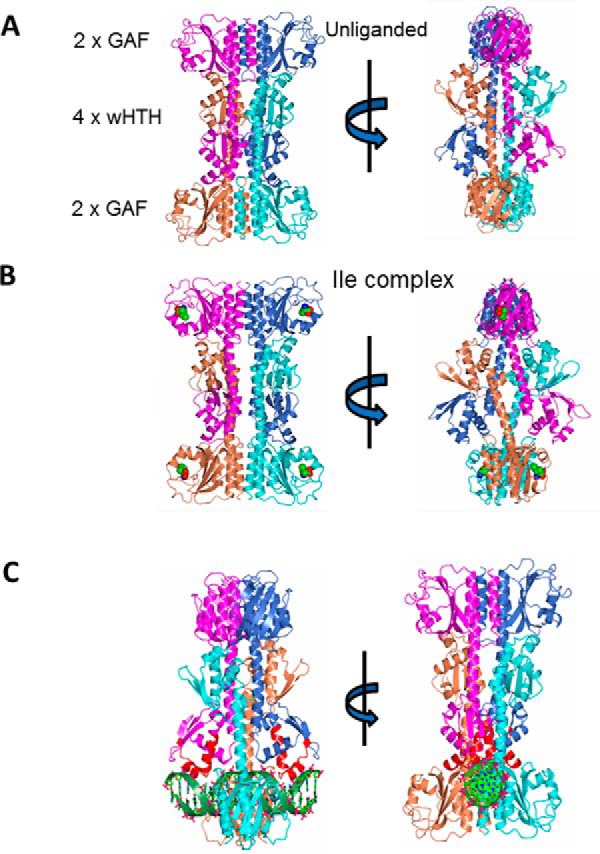
**Tetrameric structures of CodY in the crystal.** The four chains that form an obvious tetramer are shown in *ribbon* representation and with chains A–D colored *magenta, light blue, cyan,* and *coral*, respectively. *A,* unliganded CodY is shown in two approximately orthogonal orientations following rotation about a vertical axis. The two pairs of GAF domains flanking the four central wHTH domains are evident. *B,* isoleucine liganded CodY(L3S) represented as above. The amino acid effector bound to each of the four GAF domains is shown as van der Waals spheres colored by atom type as follows: carbon, *green*; oxygen, *red*; nitrogen, *blue. C,* modeled DNA complex of the unliganded CodY tetramer observed in the crystal, illustrating the steric clash between the DNA duplex and the distal pair of GAF domains. The duplex DNA is shown as a *ribbon* backbone (*green*), and the nucleic acid atoms are shown in *ball-and-stick* and colored by atom type. The DNA molecule has been juxtaposed with the CodY protomers using rigid body superposition methods so that the HTH (colored *red*) and wing elements of the wHTH domains in the proposed AB dimer fit into the major and minor groove of DNA as observed in the structure of the FadR-DNA complex (PDB code 1h9t). In these orthogonal views it is evident that the GAF domains from chains C and D clash sterically with the DNA.

The tetramer has approximate overall dimensions of 105 × 75 × 75 Å (Fig. 2). The GAF domains exist as two pairs of dimers flanking four wHTH domains. The latter are centrally located and tetrahedrally arranged. Each GAF domain dimer has a 2-fold axis of symmetry running in the vertical direction in Fig. 2. The GAF domain dimer is formed by interactions of helices α1 and α5 in subunits A and B in Fig. 2, creating an intermolecular helical bundle. Equivalent interactions are formed for subunits C and D. The central sections of the α5 helices in each dimer extend in the vertical direction from the GAF domains to form interactions with the pair of wHTH domains emerging from the distal pair of CodY protomers. Finally, these helices then extend into the wHTH domain where residues 165–177 of all four chains contribute to a second intermolecular helical bundle. A local hydrophobic core is created by the packing of the side chains of Ala-170, Val-171, and Met-174 from each of the four subunits. There is, in addition, the opportunity for polar interactions between the side chains of Lys-169 and Gln-173. A similar but not identical tetrahedral organization of four wHTH domains was observed in the crystal structure of CodY(168–259) determined previously (Protein Data Bank code 1b18) (23); these quaternary interactions were considered to be without physiological significance, in part because these domains possessed an artificial N terminus (23).

The domain topology of the isoleucine-bound CodY(L3S) tetramer is superficially similar to that of the unliganded wild-type form; however, the packing of the chains is significantly different (Fig. 2*B*). As shown in the *right-hand panels* of Fig. 2, the lateral protrusion of the wHTH domains is more striking in the liganded form. This results from changes in the relative orientations of the long interdomain helices, as discussed later. For both the liganded and unliganded CodY tetramers, although the wing elements of the wHTH are on the outside of the assembly, the so-called recognition helices of the HTH (α8) are closer to the center of the tetramer and residues from the turn connecting the two helices of the HTH are in close proximity to the GAF domains of the distal subunits. As a result, in both the unliganded CodY and isoleucine-bound CodY(L3S) tetramers, DNA is sterically hindered from binding to the wHTH domains (Fig. 2*C*).

##### SEC-MALLS Reveals a CodY Dimer

**FIGURE 3.**
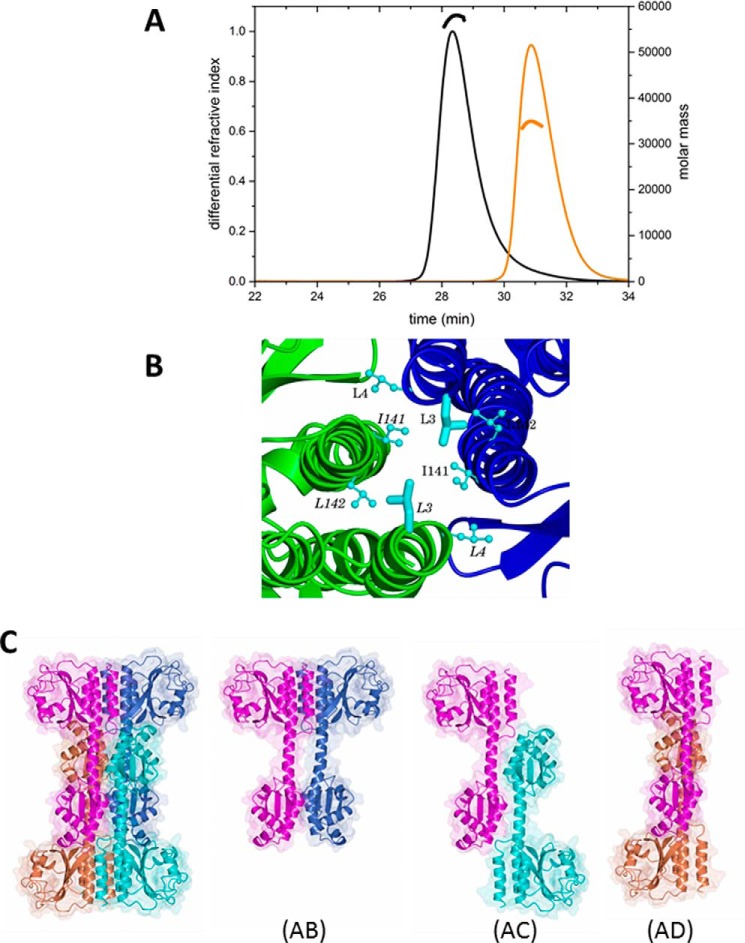
**Quaternary structure of CodY.** *A,* molecular mass measured from SEC-MALLS analysis. 100 μl of 1 mg·ml^−1^ protein samples were loaded onto a Superdex S200 10/300 gel filtration column. The *continuous thin lines* trace the refractive index of the eluate from this column as a function of time. The *short thicker lines* represent the weight average molecular weight of the species in the eluate calculated using the light-scattering measurements. The *black lines* show the data from the experiment with wild-type CodY, and the *orange lines* show the data for CodY(L3S). *B,* GAF domain interface of chains A and B in the dimer. The contributions of the pair of Leu-3 residues (side chains shown as *cylinders*) to the local hydrophobic core, through interactions with Leu-4, Ile-141, and Leu-142 residues (side chains shown as *ball-and-stick*), are evident. *C,* possible dimers arising from the CodY tetramer. The unliganded CodY tetramer, colored by chain, is shown on the *left* with the three possible dimers AB, AC, and AD shown moving from *left to right*.

Because CodY is thought to be a dimer (23, 25), the observation of tetramers in the crystals was unexpected even though earlier gel filtration data had shown evidence for a possible dimer-tetramer equilibrium (26). We therefore re-investigated the quaternary structure of CodY by performing a SEC-MALLS analysis. In these experiments, CodY (1 mg·ml^−1^) samples were incubated in the presence and absence of isoleucine (10 mm) and passed down a Superdex S200 column. As the material eluted from the column, the refractive index was recorded to give a measure of protein concentration, and the light scattering was recorded to allow calculation of the molar mass. As shown in Fig. 3*A*, CodY elutes as a single symmetric peak in these experiments, and the molecular mass calculations reveal a stable species of 58 kDa. CodY has 259 amino acid residues and a calculated mass of 29 kDa, and we conclude that the protein is dimeric under these solution conditions. The inclusion of isoleucine in the column chromatography buffer had no effect on the trace. These experiments show that CodY is a dimer at a concentration of 1 mg·ml^−1^ (∼35 μm) in solution. In contrast, at the 10–20-fold higher CodY concentrations used for crystallization, CodY is evidently able to form tetramers. The intracellular concentration of CodY would be ∼3 μm if we make a generous estimate of 10,000 CodY protomers per *B. subtilis* cell (30). We conclude that CodY is a dimer under physiological conditions.

##### CodY(L3S) Is Predominantly Monomeric

These SEC-MALLS experiments were repeated for the CodY(L3S) mutant. The profiles revealed a lower molecular mass species of ∼34 kDa indicating that this protein is predominantly monomeric (Fig. 3*A*). Leu-3 is situated in the interface between the GAF domains in the tetrameric assembly where it forms interactions with the side chains of Leu-3 and Ile-141 in the partner subunit that contribute to a local intermolecular hydrophobic core (Fig. 3*B*). Substitution of the Leu-3 residues with smaller and polar serines would be expected to destabilize this interface. However, at higher protein concentrations, it is evident that CodY(L3S) assembles to form dimers as well as the tetramers that are observed in the crystals.

##### CodY(L3S) Is Functional in Vivo

As the Leu-3 to Ser substitution is present in the crystals of the CodY-isoleucine complex and because this mutation affects the stability of CodY dimers, we constructed a *B. subtilis* strain harboring a version of *codY* encoding the L3S mutation. The *codY*(*L3S*) mutant was introduced into *B. subtilis* strains that harbor at the *amyE* locus a transcriptional *lacZ* fusion to the promoters for either *ybgE* or *bcaP* (15, 31). *P*_bcaP_ and *P*_ybgE_ are known to be regulated by CodY in wild-type *B. subtilis* (15, 29). β-Galactosidase activity was measured in the *codY*(*L3S*) strain and in otherwise isogenic wild-type *codY* and *codY* null mutant strains. In a defined medium in which CodY is maximally active, β-galactosidase activity dependent on the *P*_bcaP_ and *P*_ybgE_ promoters in the *codY*(*L3S*) strain was 100- and 20-fold higher, respectively, than in the wild-type strain (Table 2). However, the level of β-galactosidase activity in the *codY*(*L3S*) strain remained 10- and 20-fold lower, respectively, than that in the *codY* null mutant strain (Table 2). This suggests that CodY(L3S) retains the capacity to bind to DNA and repress transcription *in vivo*, even though its activity is considerably lower than that of the native CodY; as a result, there is significant derepression of transcription of the target genes. This result suggests that weakening of the dimer-forming interactions between the GAF domains lowers CodY activity consistent with the notion that CodY functions as a dimer in *B. subtilis* cells.

**TABLE 2:** **Transcriptional fusion data**

Strain	Genotype	β-Galactosidase activity
**Expression of the *bcaP-lacZ* fusion in TSS + 16-amino acid medium**		
BB2505	WT	0.15
BB3663	*codY*(*L3S*)	14.2
BB2548	*codY*	137.2

**Expression of the *ybgE-lacZ* fusion in TSS + 16-amino acid medium**		
BB2770	WT	1.12
BB3669	*codY*(*L3S*)	23.6
BB2771	*codY*	428.0

##### CodY Dimer Is Most Likely Formed through GAF-GAF Domain Interactions

The observation of dimers of wild-type CodY in the SEC-MALLS experiment prompted us to consider which of the pairs of chains in the tetramer observed in the crystal constitute the dimer observed in solution. There are three possibilities as illustrated in Fig. 3*C,* AB (equivalent to CD), AC (equivalent to BD), and AD (equivalent to BC). The interfacial areas in the three possible dimers are 830 Å^2^ (AB), 720 Å^2^ (AC), and 1300 Å^2^ (AD) in the unliganded protein and 830 Å^2^ (AB), 130 Å^2^ (AC), and 1410 Å^2^ (AD) in the CodY(L3S) isoleucine complex.

We favor the AB dimer for three reasons. First, the AB dimer preserves the interface observed in the structure of the isolated GAF domain dimer, and the residues of this interface are well conserved among CodY orthologues (23). Moreover, much of the surface of the protein that contributes to this interface is hydrophobic. Thus, the exposure of this surface in the AC and AD dimers would be expected to be energetically unfavorable. Second, the L3S mutation, which disrupts CodY dimer formation, maps to the GAF-GAF interface, which is unique to the AB dimer. Finally, simple modeling shows that the wHTH elements in the AB dimer would be able to bind to DNA in a conventional manner. In contrast, the AC and AD dimers would be severely sterically hindered from doing so by the presence of the distal GAF domains. We conclude that the AB dimer is most likely to be the form observed in solution by SEC-MALS.

##### Structural Changes Accompanying Ligand Binding

As stated previously, we were unable to obtain crystals of liganded wild-type CodY or unliganded CodY(L3S). Therefore, to explore the basis of branched-chain amino acid regulation of DNA binding, we compared the three-dimensional structures of the unliganded wild-type CodY and liganded CodY(L3S) forms, mindful of the caveats associated with the L3S mutation.

**FIGURE 4.**
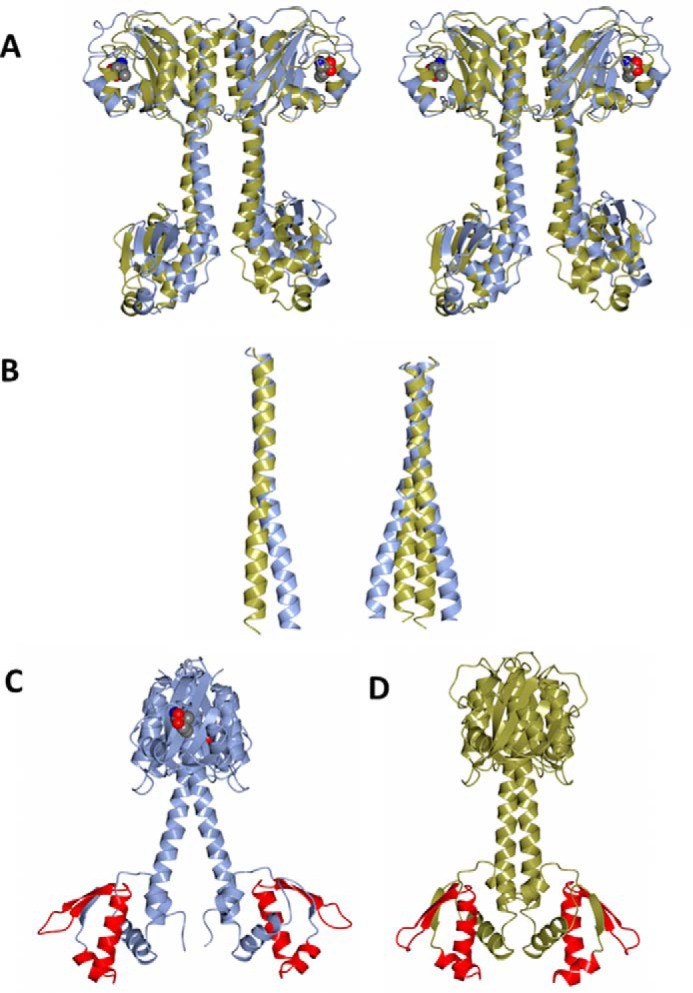
**Structural changes in the CodY dimer upon ligand binding.** *A,* stereo view of the unliganded CodY dimer (*gold*) and the isoleucine-liganded CodY(L3S) dimer (*blue*) following superposition of the Cα atoms from their GAF domains. The isoleucine ligand is shown as van der Waals spheres colored by element. *B,* long interdomain helices following superposition of the unliganded CodY dimer (*gold*) and the isoleucine-liganded CodY(L3S) (*blue*) as in *A*. These are shown for a single subunit (*left*) where the changing course of this α-helix is observed, and for the dimer (*right*) where the splaying of the pair of helices can be seen. *C* and *D,* overall structures of the liganded CodY(L3S) with chains colored *blue* in *C* and unliganded CodY with chains colored *gold* in *D*. The orientation of the dimers in *C* and *D* is approximately orthogonal to that in *A*. The splaying of the long helices and the increased separation of the wHTH domains accompanying isoleucine binding can be seen. Residues 203–240 spanning the helix-turn-helix and the associated wing, consisting of a loop and flanking β-strands, are shown in *red*.

Thus, the unliganded CodY and liganded CodY(L3S) dimers were superposed by least squares minimization procedures applied to the positions of the backbone Cα atoms of the GAF domains from the AB dimers (Fig. 4). This gives a positional r.m.s.Δ of 3.5 Å for 304 equivalent Cα atoms. However, 270 of these Cα atom pairs can be superposed with an r.m.s.Δ of 1.3 Å, emphasizing that very large structural changes in the GAF domains are localized to the site of isoleucine binding (Fig. 4*A*). Following this superposition, it is apparent that isoleucine binding to CodY(L3S) is associated with displacements in the long α5 helix and a significant separation of the two wHTH domains in the dimer (Fig. 4). This results from changes in the directions of the interdomain helices α5 as they emerge from the GAF domains (Fig. 4*B*). The structural changes can be described as a combination of bending and twisting motions (Fig. 4*A*). In the dimer, the structural changes in each subunit lead to a significant splaying of the two helices and a noticeable separation of the wHTH domains. wHTH domains typically bind to DNA by aligning their recognition helices with the major groove of the DNA, with their wing elements forming contacts with the ribose-phosphate backbone and in some instances extending into the adjacent minor groove. Dimeric wHTH domain-containing DNA-binding proteins often bind to recognition sequences with elements of 2-fold symmetry, often referred to as palindromic, that match the 2-fold symmetry of the protein itself. The distance of separation of the wHTH domains is an important determinant of DNA binding, so that the alteration in the juxtaposition of the DNA binding domains of CodY upon ligand binding would be expected to change the ability of CodY to bind to DNA. At the resolution of the structures determined here, however, it is difficult to discern how changes in the GAF domain upon ligand binding lead to changes in helix α5 and the relative arrangement of the wHTH domains.

##### DNA Binding

A 15-nucleotide consensus sequence AATTTTC*W*GAAAATT (where *W* = A or T) that was first identified for the CodY protein from *L. lactis* (18, 19) has been shown to play a crucial role in *B. subtilis* CodY function (20). To provide reagents for co-crystallization of CodY with DNA and to further analyze CodY binding, short oligonucleotide duplexes of 19, 31, or 36 bp were designed based on the upstream CodY-binding motif of the *bcaP* promoter (31). The 19-, 31-, and 36-bp constructs consist of the 15-bp consensus motif with 2 downstream bp and 2, 14, or 19 upstream bp, respectively (Table 3). In each case, three nucleotide substitutions (the p8 mutation; underlined in Table 3) (31) were introduced into the CodY-binding motif to generate a perfect consensus sequence. Although crystals containing both DNA and protein were obtained from crystallization experiments set up with mixtures of CodY and the BcaPp8(36) duplex, these did not diffract.

**TABLE 3:** **Oligonucleotides used in DNA binding**

DNA (source)*^a^*	Sequence*^b^*
**BcaPp8(19) (*P_bcaP_*-56 to-38)**	
oBB459	5′-CG**AATTTTCTGAAAATT**TT
oBB460	5′-AA**AATTTTCAGAAAATT**CG

**BcaPp18(31)** (***P_bcaP_*-68 to-38)**	
oBB527	5′-gActgAcTgacTCG**AATTTTCTGAAAATTTT**
oBB528	5′-AA**AATTTTCAGAAAATT**CGAgtcAgTcagTc

**BcaPp8(36) (*P_bcaP_*-73 to-38)**	
oVML12	5′-CAAAATAAAAAATTTGTCG**AATTTTCTGAAAATT**TT
oVML13	5′-AA**AATTTTCAGAAAATT**CGACAAATTTTTTATTTTG

**BcaPp8(31) (*P_bcaP_*-68 to-38)**	
oBB463	5′-TAAAAAATTTGTCG**AATTTTCTGAAAATT**TT
oBB464	5′-AA**AATTTTCAGAAAATT**CGACAAATTTTTTA

*^a^* The primer pairs in each box are complementary oligonucleotides that were mixed to generate duplexes used in the DNA binding experiments.

*^b^* Bold type in the sequence indicates the region with a match to the CodY consensus sequence. Underlining denotes a substitution in the sequence to match the consensus more closely. Lowercase letters indicate positions where the BcaPp18(31) duplex differs from the BcaPp8(31) duplex.

**FIGURE 5.**
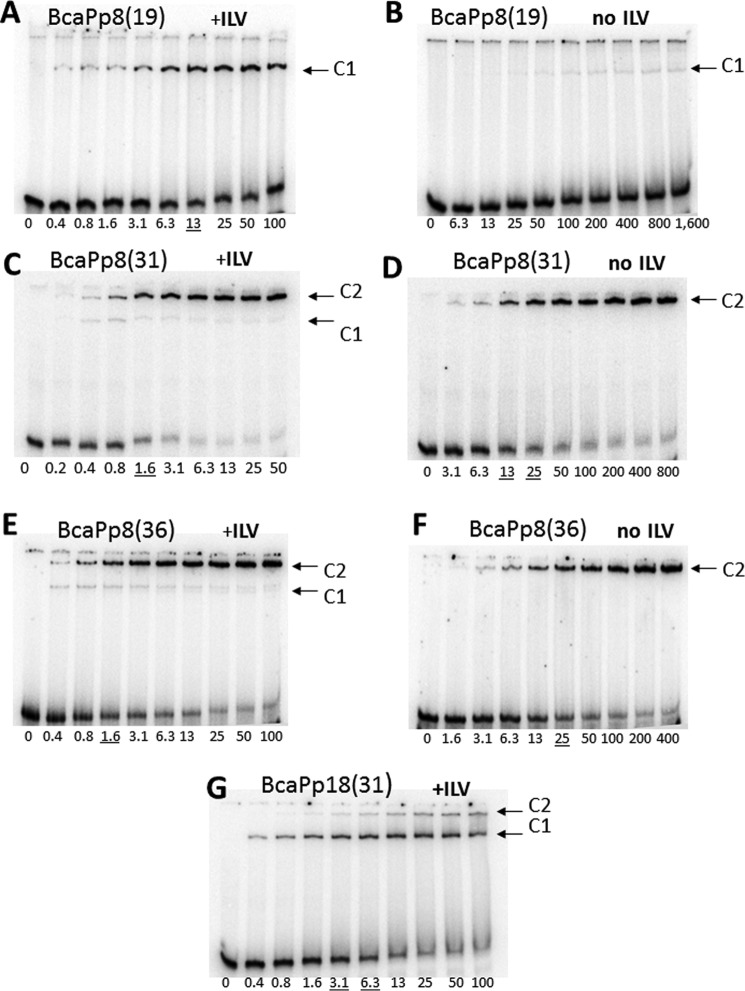
**Gel electrophoretic mobility shift assay of CodY binding to *bcaP* promoter fragments.** Labeled BcaPp8(19) (*A* and *B*), BcaPp8(31) (*C* and *D*), and BcaPp8(36) (*E* and *F*) were incubated with increasing amounts of purified CodY in the presence (*A, C,* and *E*) or absence (*B, D,* and *E*) of 10 mm isoleucine/leucine/valine. The CodY concentrations used (nanomolar of monomer) are indicated *below* each lane. The *underlined* values indicate the concentration of CodY needed to shift 50% of the DNA fragments (under conditions of vast CodY molar excess). The *arrows* indicate the complexes C1 and C2 described in the text. *G,* assay of CodY binding to the BcaPp18(31) duplex containing multiple mutations in one of the putative CodY-binding subsites.

In electrophoretic mobility shift assays, wild-type CodY in the presence of a mixture of isoleucine, leucine, and valine (ILV) was able to bind the BcaPp8(19) duplex, indicating that this short sequence is sufficient for protein-DNA interaction (Fig. 5*A*). Binding of wild-type CodY to the BcaPp8(31) or BcaPp8(36) duplexes was even more efficient, with an apparent *K_d_* of 1.6 nm (Fig. 5, *C* and *E*). The affinity of CodY for the longer duplexes is almost identical to its affinity for the extended 235-bp *bcaPp8* fragment (1 nm) (31), indicating that the 31-bp duplex contains all the determinants important for binding. Interestingly, CodY formed only one type of complex (C1) with the BcaPp8(19) duplex; however, an additional, lower mobility complex (C2) was the predominant form in the presence of the BcaPp8(31) or BcaPp8(36) duplexes (Fig. 5, *A*, *C,* and *E*). The two complexes are likely to represent species with different CodY-binding stoichiometries; BcaPp8(19) is presumably bound by one CodY dimer, whereas BcaPp8(31) and BcaPp8(36) are each bound by two CodY dimers. In the absence of ILV, CodY formed complexes with DNA with considerably lower affinity (Fig. 5, *B, D,* and *F*). The loss of affinity was very dramatic in the case of the BcaPp8(19) duplex. No higher mobility complex was observed with the longer duplexes, suggesting impaired stability of this complex in the absence of ILV (Fig. 5, *D* and *F*).

**FIGURE 6.**
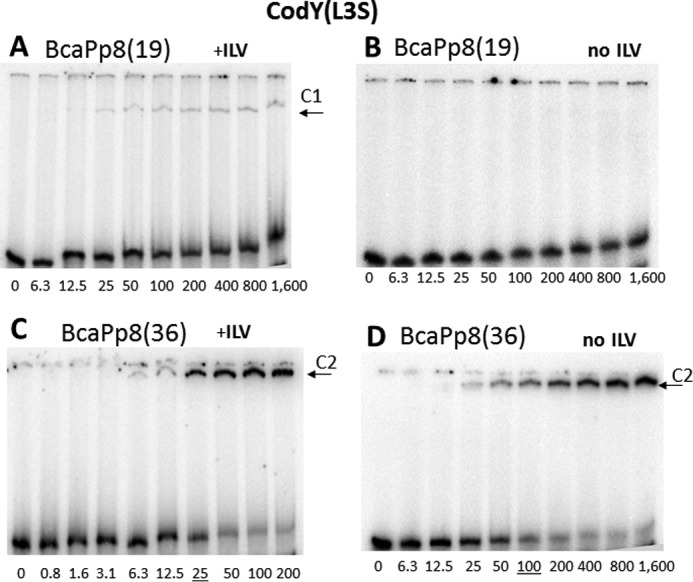
**Gel electrophoretic mobility shift assay of CodY(L3S) binding to *bcaP* promoter fragments.** Labeled BcaPp8(19) (*A* and *B*) and BcaPp8(36) (*C* and *D*) were incubated with increasing amounts of purified CodY(L3S) in the presence (*A* and *C*) or absence (*B* and *D*) of 10 mm isoleucine/leucine/valine. The CodY concentrations used (nanomolar of monomer) are indicated below each lane. The *underlined* values indicate the concentration of CodY needed to shift 50% of the DNA fragments (under conditions of vast CodY molar excess). The *arrows* indicate the complexes C1 and C2 described in the text.

Binding of CodY(L3S) to the BcaPp8(19) and BcaPp8(36) duplexes was less efficient than that of the wild-type CodY, consistent with the lower activity of the mutant protein *in vivo* (Fig. 6). CodY(L3S) bound very weakly to the BcaPp8(19) duplex even in the presence of ILV (Fig. 6, *A* and *B*). Moreover, it did not form the higher mobility complex with the BcaPp8(36) duplex (Fig. 6, *C* and *D*). The weak capacity of CodY(L3S) to form the higher mobility complex is consistent with its defect in dimer formation in solution. However, CodY(L3S) dimers are apparently stabilized if two of them are allowed to interact together, as revealed by the relatively efficient formation of the lower mobility complex with the BcaPp8(36) duplex.

**FIGURE 7.**
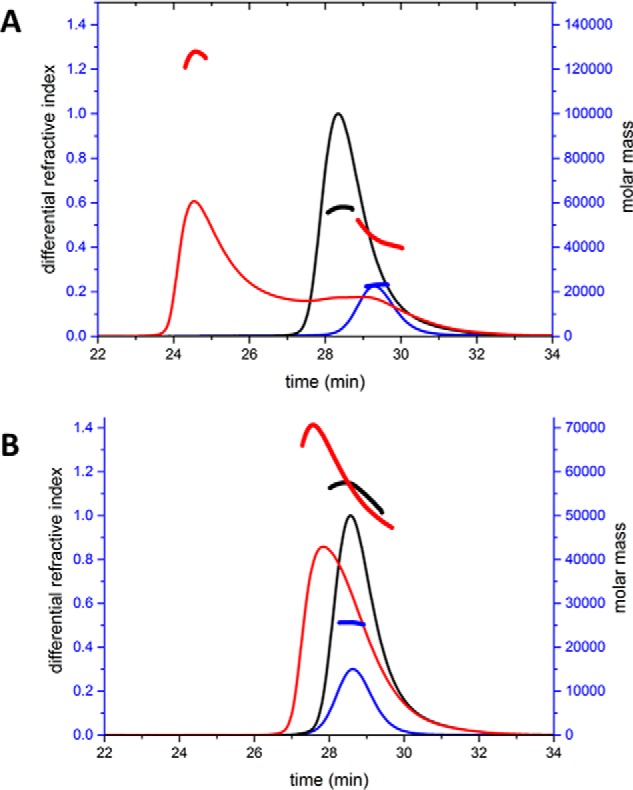
**DNA binding analyzed by SEC-MALLS.** 100-μl samples of 1 mg·ml^−1^ CodY incubated in the presence and absence of a molar equivalent of the BcaP36 oligonucleotide duplex (19 μm), and in the presence (*A*) and absence (*B*) of isoleucine (10 mm), were loaded onto a Superdex S200 10/300 gel filtration column. The *continuous lines* trace the refractive index of the eluate from this column as a function of time. The *thick shorter lines* represent the weight average molecular weight of the species in the eluate calculated using the light-scattering measurements. The *black lines* show the data from the experiments with CodY alone; the *blue lines* show the traces for the BcaP36 oligonucleotide duplex alone. The *red lines* show the data from the experiment in which CodY and BcaP36 were pre-incubated together in the presence (*A*) and absence (*B*) of isoleucine, respectively. For the experiments in which isoleucine was present, the amino acid was also included in the column equilibration and running buffer.

The interaction of CodY with the BcaPp8(36) duplex DNA was further examined using SEC-MALLS. As shown in Fig. 7, the DNA duplex eluted as a single symmetrical peak from the Superdex S200 column with an *M*_r_ value calculated from the light scattering data of 23 kDa. This is consistent with its molar mass of 22 kDa. Similarly, in the absence of DNA, the experimentally derived mass of CodY is 58 kDa, consistent with a dimer. In the two subsequent experiments, CodY was incubated with BcaPp8(36) in the presence and absence of 10 mm isoleucine. In the absence of isoleucine, we observed a somewhat asymmetric peak in the differential refractive index profile centered at 28 min (Fig. 7*B*), a lower retention time than that of CodY alone. The molar mass associated with this species is ∼70 kDa. In contrast, the presence of isoleucine caused a significant reduction in the retention time of the principal species present and a significant rise in its molecular mass to ∼130 kDa. We interpret the 130-kDa species to be a complex formed between four molecules of CodY and one BcaPp8(36) duplex (138 kDa). We observed a similar ∼130-kDa species when analyzing a mixture of CodY and a 32-bp pair duplex derived from the CodY-regulated *yurP* (*frlB*) promoter (20) in the presence of isoleucine but not in its absence (data not shown).

The 70-kDa species observed in the absence of isoleucine has a higher mass than that expected for a CodY dimer (58 kDa) but lower than that expected for a dimer of CodY bound to one BcaPp8(36) duplex (81 kDa). It can be seen that the peak on the chromatogram for this complex overlaps with the leading edge of the peak for the free DNA trace. The presence of the uncomplexed DNA would lower the weight average molecular weight of the eluting species calculated by MALLS, consistent with the presence of a higher molecular weight protein-DNA complex such as CodY_2_-BcaPp8(36). The EMSA experiments suggest, however, that this species would not be observed, and it is more likely that the profile reflects the extensive dissociation of CodY_4_-BcaPp8(36) complexes accompanying chromatography in the absence of Ile.

These results confirm that the C1 species observed in the EMSA experiments represent CodY_2_ complexes with DNA and that the lower mobility C2 species are complexes of CodY_4_ and BcaPp8(31) or BcaPp8(36), implying the presence of two CodY_2_-binding subsites on these longer duplexes. Therefore, one might expect that inactivation of one of the subsites within such a DNA duplex would reduce binding of one of the CodY dimers resulting in the formation of a DNA complex containing only one CodY dimer. Indeed, when we introduced multiple mutations in the 5′ part of the BcaPp8(31) duplex by creating its p18 version (Table 3), we observed a significant reduction of C2 complex formation and the emergence of C1 as the predominant complex with CodY (Fig. 5*G*).

## Discussion

### 

#### 

##### CodY Is a Dimer That Binds Cooperatively to bcaP DNA

The structures of full-length CodY from *B. subtilis* determined here reveal the effector binding domain and the DNA binding domain as discrete entities separated by a helical linker. In the crystals, four CodY protomers are assembled into an unusual tetramer in which four wHTH domains are sandwiched between two GAF domain dimers. For the unliganded form in particular, the tetramer is compact with extensive subunit interfaces. From within this tetramer structure, there is no possibility that the wHTH domains of CodY could bind to DNA in a conventional manner, because the GAF domains of partner subunits in the tetramer occupy a substantial volume that would be taken up by the DNA duplex (Fig. 2*C*). We therefore considered that this tetrameric form might represent an inactive state of CodY that prevails in the absence of nutritional signals. However, we found no evidence for CodY tetramers in SEC-MALLS experiments, which conclusively showed that CodY is a dimer even at protein concentrations higher than those found *in vivo* (Fig. 3*A*). Furthermore, unliganded CodY can bind to DNA, albeit with lower affinity than the BCAA-liganded protein (Fig. 5). Thus, there is no evidence to suggest that the CodY tetramers observed here by crystallography are physiologically relevant. There are many cases where plausible, stable interfaces between molecules in crystals prove to have no functional significance, including examples from studies of proteins involved in gene regulation in *Bacillus* (32, 33).

**FIGURE 8.**
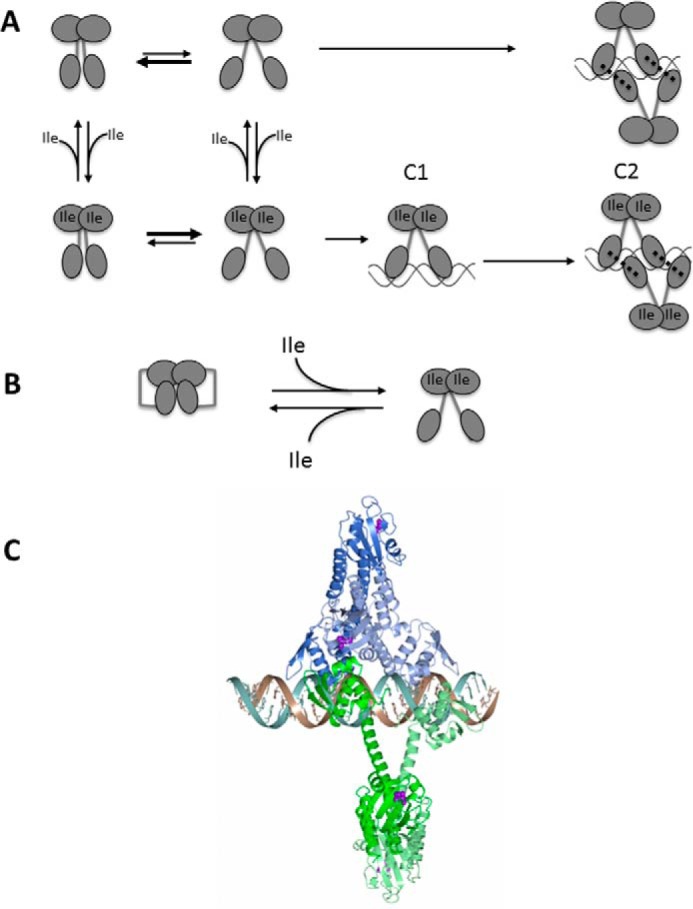
**Models for CodY action.** *A,* model for CodY binding to *bcaP* promoter DNA fragments in the EMSA experiments. The model shows complexes with CodY protomer/DNA duplex stoichiometries of 2:1 and 4:1. The thickness of the *arrows* indicates the direction of the equilibrium. In this model, splaying is needed for DNA binding, and the proportion of dimers in the splayed state is controlled by the BCAA, isoleucine. The splayed conformation in this schematic may or may not correspond to that in the crystal structure of CodY(L3S)-Ile. The *dashed lines* indicate non-covalent interactions between a pair of CodY dimers bound to DNA that would give rise to cooperative binding. *B,* model of ligand-dependent activation in CodY in which non-covalent interactions between the wHTH and GAF domains within each protomer of an unliganded CodY dimer are disrupted upon isoleucine binding. *C,* structural model for the CodY_4_-DNA complex in which each CodY chain is shown as a *ribbon* and colored by dimer. The two CodY dimers are shown bound to opposite faces of the DNA duplex with “interdigitation” of the wHTH elements from each dimer.

An interesting and important finding from this work is the establishment that CodY forms alternate complexes with short DNA fragments derived from the *bcaP* promoter. With the shorter 19-bp duplex, CodY forms a single complex. With the 31- and 36-bp duplexes, it forms two complexes as shown schematically in Fig. 8*A*. The first, C1, has a mobility similar to that of the complex formed with the 19-mer, whereas the second predominant complex, C2, has lower electrophoretic mobility consistent with a larger size (Fig. 5). SEC-MALLS analysis of CodY mixed with BcaPp8(36) in the presence of isoleucine showed the formation of a complex consistent with four CodY protomers bound to a single duplex (Fig. 7). Collectively, our data suggest that C1 is CodY_2_-BcaPp8 with one CodY dimer bound per duplex and that C2 is CodY_4_-BcaPp8 with two dimers per duplex. Interestingly, in the absence of branched chain amino acids, the C1 complex appears to be much less stable for BcaPp8(19) and is not discernible at all for BcaPp8(31) or BcaPp8(36) (Fig. 5*D*). These data are consistent with cooperative binding of CodY to the longer duplexes with the affinity of the second dimer for the DNA augmented by the binding of the first dimer. Cooperativity, exhibited in the presence and the absence of ILV, may arise because the binding of the first CodY dimer to the DNA induces a conformational change in the duplex such that the second dimer can bind more readily. In this regard, the *bcaP* promoter fragments used here have a high bending propensity (34). Alternatively, the binding of the first dimer may help in the recruitment of the second dimer by contributing protein-protein interactions to supplement the protein-DNA interactions (Fig. 8*A*). In the case of CodY, these interdimer interactions may be contributed either by the GAF domains or, as shown schematically in Fig. 8*A*, by the wHTH domains.

The exact role of protein-protein interactions in binding of two dimers and the precise contribution of each of the two subsites (Fig. 8*A*) to CodY binding remain unknown. It is also not known whether all CodY-binding sites are composed of two (or more) subsites; the exact stoichiometry of CodY binding to all CodY-binding sites may be not identical.

##### Structural Implications for DNA Binding

Comparisons of the structures of unliganded CodY and effector-bound CodY(L3S) reveal striking differences in the relative positions of the wHTH domains (Fig. 4). These differences could reflect the effect of Ile binding on CodY conformation, which would lead us to conclude that the splayed conformation of CodY is more conducive to DNA binding, because Ile-bound CodY has a higher affinity for all *bona fide* binding sites tested to date. This conclusion relies, however, on the assumption that the mode of DNA binding is same for CodY(L3S) and wild-type CodY bound to Ile.

**FIGURE 9.**
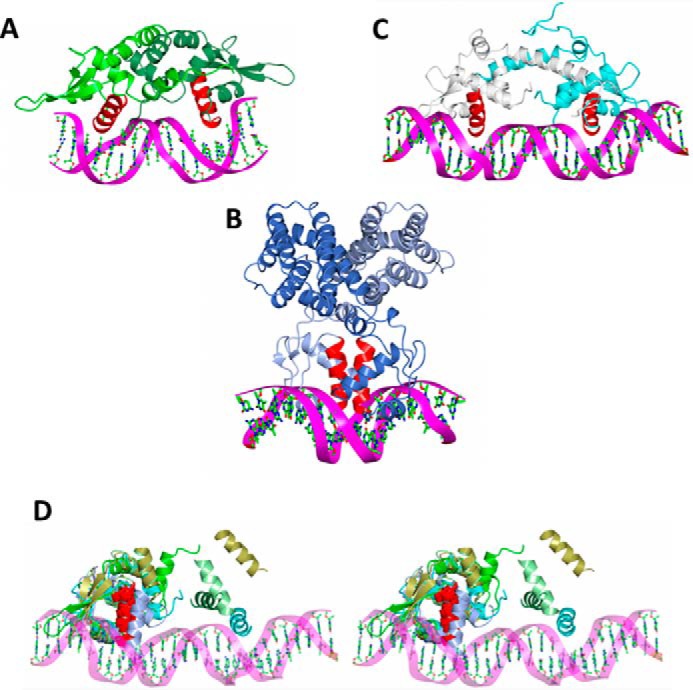
**Comparison of homodimeric wHTH repressors with CodY.** The QsrR-DNA complex (*A*) with subunits colored in different shades of *green* (35), the FadR-DNA complex (36) (*B*) with subunits colored *ice-blue* and *light blue,* and the IscR-DNA (37) (*C*) complex with chains colored *cyan* and *white* are shown. In each image the recognition helices are colored in *red. D,* stereo image showing the superimposed wHTH domains from the A chains of QsrR (*bright green*), FadR (*ice-blue*), IscR (*cyan*), unliganded CodY (*light green*), and CodY(L3S)-Ile (*gold*) in the context of the IscR operator DNA. As before, the recognition helix of the A subunit is colored in *red*. The recognition helix from the partner subunit is shown colored according to structure as before. The differences in the juxtaposition of the pair of recognition helices in the various dimers are apparent.

To assess the implications of the CodY structures for DNA binding, we analyzed other homodimeric wHTH domain-containing transcription factors, whose structures have been solved in complex with DNA. The most closely similar structure is of the quinone-sensing response regulator QsrR from *S. aureus,* whose subunits can be overlaid on the wHTH domain of CodY with an r.m.s.Δ of 2.9 Å for 85 aligned residues giving a *Z*-score of 4.1 (35). In the complex of QsrR with a 17-bp duplex, the respective recognition helices bind in consecutive major grooves of the DNA. These α-helices are related by a 28-Å translation and a 70° rotation (Fig. 9*A*). Binding to the operator DNA in this system is reversed upon covalent attachment of menadione to Cys-5 of QsrR, which leads to a 10° rotation and 9 Å separation of the subunits (35). Another structurally well characterized example is FadR of *Escherichia coli,* which regulates genes encoding enzymes of fatty acid metabolism. In the structure of its complex with a 19-bp *fadB* operator DNA, the two recognition helices of the wHTH domains are closely abutting (their helix axes can be superposed following a 9 Å translation and a 30° rotation) in the major groove of the DNA giving rise to a compact protein DNA complex (Fig. 9*B*). Binding of the effector, myristoyl-CoA, to the N-terminal domain leads to separation of the recognition helices by a further 7 Å, loss of DNA binding, and relief of repression (36). In IscR, which regulates genes involved in iron-sulfur cluster biogenesis, longer DNA recognition sequences spanning 26–29 bp were used in crystallization (Fig. 9*C*) (37, 38). In these complexes, the wHTH domains are separated by long C-terminal helices that mediate dimer formation such that the recognition helices of the wHTH in the respective subunits are related by a displacement of 36 Å and a 71° rotation. In this system, the iron-sulfur cluster effector binds within the wHTH itself so as to change the promoter specificity of the repressor.

To facilitate comparison, we superimposed the wHTH domains of one of the chains (chain A) of unliganded CodY and Ile-bound CodY(L3S) onto one of the corresponding domains of QsrR, FadR, and IscR in their DNA complexes and observed the juxtaposition of the recognition helix in the partner wHTH domains and the DNA (Fig. 9*D*). This simple modeling suggests that the unliganded CodY dimer, in which the recognition helices from each chain are related by a 25 Å translation and a 40° rotation, could bind to a DNA duplex of ∼20 bp. For the Ile-bound CodY(L3S) structure, where splaying increases the translational and rotational separation of the recognition helices to 34 Å and 45°, a longer DNA recognition sequence of ∼30 bp would be needed.

This analysis indicates that 19 bp is just sufficient to bind a CodY dimer in the more compact conformation we observe in the unliganded CodY crystals. Meanwhile, a pair of CodY dimers in the splayed conformation could bind to the 31-mer (BcaPp8(31)) and 36-mer (BcaPp8(36)) DNA duplexes only if the binding sites for each dimer are significantly overlapping as depicted in Fig. 8*A* and modeled in Fig. 8*C*. Such a mode of binding has been proposed previously (39). However, the modeling predicts that the splayed CodY dimer would be unable to bind to the 19-mer, BcaPp8(19). This is consistent with the poor ability of CodY(L3S) to bind the BcaPp8(19) duplex but is contrary to the experimental data obtained with the wild-type CodY. One explanation for this is that the splayed conformation of CodY obtained with CodY(L3S) is specific to the mutant form of the protein. Alternatively, the CodY dimer may be able to engage the 19-mer duplex using just a single wHTH domain.

##### Concluding Remarks

Crystals of CodY grew irreproducibly perhaps due to inherent flexibility in the CodY dimer, arising from motions of the wHTH domains relative to each other and the GAF domains. At low frequency, tetramers are evidently assembled in which these domain motions are “frozen out” by dimer-dimer interactions, and this overcomes the barrier to lattice formation. A precedent for this is calmodulin, where early structures revealed independent calcium binding domains connected by a long α-helix in a dumb-bell arrangement (40). Later structures of calmodulin-target protein complexes showed altered conformations of the linker region and a spectrum of more compact relative domain arrangements (41, 42).

The helical conformation of residues 155–168, which link the two domains in CodY, is evidently stabilized by quaternary interactions with the distal pair of wHTH domains in the tetramers within the crystal. In the absence of these stabilizing interactions, these residues could take up a different conformation or range of conformations. In this regard, the string of five glutamate side chains in six residues (160–165, Fig. 1) in adjacent linker regions in the dimer would be expected to destabilize electrostatically the compact helical conformation in this region of the subunit, possibly giving rise to a more extended conformation in solution. Interestingly, it has been shown for some promoters that the extent of CodY binding to DNA and CodY activation by isoleucine is pH-dependent (39). This behavior may be associated with the titration of one or more of these glutamates. Regardless, this flexibility could allow the wHTH domains to be brought into contact with the GAF domains within the dimer so that they are able to sense directly the conformational changes brought about by BCAA binding as shown schematically in Fig. 8*B*. This would provide a basis for explaining the puzzling absence in the crystal structures of an obvious route for transducing the substantial structural changes upon ligand binding to the GAF domain, into structural alterations of the wHTH domains. A rigorous elucidation of the structural basis of (i) effector control of CodY activity and (ii) CodY recognition of the 15-bp consensus DNA-binding motif, with its considerable degeneracy but clear 2-fold symmetry, awaits the determination of structures of CodY-DNA complexes.

While this manuscript was under review, crystal structures of CodY from *Bacillus cereus* (*Bc*CodY) and *S. aureus* (*Sa*CodY) were reported (43). The domain arrangement in these orthologous proteins is similar to that observed in CodY from *B. subtilis*, and tetramers similar to those reported here were observed for *Bc*CodY. Curiously, the BCAA-binding site in unliganded *Bc*CodY resembles that of the liganded CodY from *B. subtilis*. Most interestingly, the *Sa*CodY structure contains GTP that is bound at the junction of the long helix and the GAF domain in a dimeric form of the protein. The authors combine their structures to propose a model in which tetramers represent a physiologically inactive state of CodY. This model has yet to be rigorously tested and is unlikely to apply to CodY from *B. subtilis*, which is clearly dimeric under conditions found in the cell.

## Experimental Procedures

### 

#### 

##### Bacterial Strains and Culture Media

The *B. subtilis* strains used in this study are described in the text and were all derivatives of strain SMY (44) and grown at 37 °C in TSS, 0.5% glucose, 0.2% NH_4_Cl minimal medium supplemented with a mixture of 16 amino acids (45, 46). The mixture contained all of the amino acids commonly found in proteins except for glutamine, asparagine, histidine, and tyrosine; the concentrations of isoleucine, leucine, and valine were 200 μg/ml each. DS nutrient broth medium with addition of agar was used for growth of bacteria on plates (46). *E. coli* strain JM107 (47) was used for isolation of plasmids and was grown in LB broth or on LB agar plates (48). The following antibiotics were used when appropriate: tetracycline, 15 μg/ml; spectinomycin, 50 μg/ml; or the combination of erythromycin, 0.5 μg/ml, and lincomycin, 12.5 μg/ml, for *B. subtilis* strains; ampicillin, 50 μg/ml, for *E. coli* strains.

##### DNA Manipulations

Methods for general DNA manipulation, transformation, DNA sequencing, gel shift assays, and sequence analysis were as described previously (20, 49). Oligonucleotides used in this work are described in Table 2. Chromosomal DNA of *B. subtilis* strain SMY or plasmid DNA was used as template for PCR as appropriate. All cloned PCR-generated fragments were verified by sequencing.

##### CodY Expression and Purification

CodY was initially produced using the pBAD30 (50) derivative plasmid, pKT1 (26), encoding residues Ala-2–His-259 of CodY with a C-terminal pentahistidine tag, with the protein purified as described previously (26). This protein was also used for the EMSA studies together with CodY(L3S)-His_5_ produced using the plasmid pBB1737, which was created by cloning the PCR fragment, synthesized using oligonucleotides oBB569 and oRPS33, into pBAD30 as described previously (27).

Two other constructs were used to overproduce CodY for the structural studies described in this work. In an attempt to obtain better diffracting crystals, we switched the polyhistidine tag to the N terminus so that it could be cleaved off after the affinity purification step. This second construct was generated by amplifying the CodY coding sequence of pBAD30-CodY using the primers CodYF1 and CodYR1 and capturing the NdeI/BamHI fragment in similarly cut pET28a(+). In addition to adding a purification tag to the encoded protein, the forward oligonucleotide introduced a leucine codon in place of a serine codon at position 3, while creating an NdeI site that facilitated cloning. The recombinant plasmid was introduced into *E. coli* BL21(DE3) for protein production. Cells were shaken in 0.5 liters of autoinduction medium for 24 h at 30 °C before harvesting by centrifugation, resuspension in 20 mm sodium phosphate buffer, pH 7.5, 0.5 m NaCl, 30 mm imidazole (Buffer A) supplemented with 1 mm 4-(2-aminoethyl)benzenesulfonyl fluoride, and lysis by sonication. Following clarification by centrifugation, the supernatant was loaded onto a 1-ml HisTrap HP column that had been pre-charged with Ni^2+^ ions and equilibrated in Buffer A. The column was washed extensively with Buffer A and developed with a 30 mm to 0.5 m linear imidazole gradient in Buffer A. After the nickel chelation chromatography step, the protein was exchanged into 20 mm Tris-HCl buffer, pH 7.5, with 50 mm NaCl and treated overnight with 1 unit/mg thrombin (BD Biosciences) to remove the polyhistidine tag and generate CodY protein with a residual tripeptide tag (Gly-Ser-His) at the N terminus and a leucine 3 to serine substitution. This CodY(L3S) protein was further purified by gel filtration chromatography on a Superdex S200 16/60 column.

A third construct was generated to direct the production of CodY, without the Leu-3 substitution and with an N-terminal human rhinovirus 3C protease-cleavable polyhistidine tag. Following polymerase chain reaction using the primers oVLC01 and oVLC02 (Table 4) and the pBAD30-CodY template, the amplified DNA was inserted into the vector pET-YSBLIC3C using ligation-independent cloning methods (51), and *E. coli* NovaBlue cells harboring a suitable recombinant plasmid were identified among kanamycin-resistant transformants. Following sequencing to confirm its authenticity, pET-YSBLIC-BsCodY was introduced in *E. coli* BL21(DE3) for protein production. The recombinant protein was purified as described above except that the polyhistidine tag was removed by overnight cleavage with human rhinovirus 3C protease. A second nickel column was run to separate the cleaved protein from the uncleaved protein and the cleaved tag. The flow-through fraction was collected, buffer exchanged into 20 mm Tris-HCl, 100 mm NaCl, and loaded onto a heparin column that was developed in this buffer over a 100–500 mm NaCl. The protein was then fractionated by gel filtration on a Superdex 200 column and concentrated by centrifugal membrane filtration using a 10-kDa molecular mass cutoff membrane filter (Amicon**[**regs]), to yield CodY with an N-terminal Gly-Pro-Ala tag in 20 mm Tris-HCl, 100 mm NaCl.

**TABLE 4:** **PCR oligonucleotide primers used in this work**

Primer	Sequence
oVML01	5′-CATGGCTAGCTTACAAAAAACAAGAATTATTAACTCCATG
oVML02	5′-TTCTGCATCCTATTAATGAGATTTTAGATTTTCTAATTCAATTAGG
oVLC01	5′-CCAGGGACCAGCAATGGCTTTATTACAAAAAACAAGAATTATTAACTC
oVLC02	5′-GAGGAGAAGGCGCGTTAATGAGATTTTAGATTTTCTAATTCAATTAG
oBB569	5′-AATTCGAGCTCAGGAGGATTATTTATCatggcttCattac
oRPS33	5′-CCCCGCATGCTTAATGATGATGATGATGATGAGATTTTAGATTTTCT-AATTCAATTAGG
oBB460	5′-AAAATTTTCAGAAAATTcg
oBB466	5′-CAAAATAAAAAATTTGTCG
oBB594	5′-gcgtttcTAgattcgtgtagaactg
oBB595	5′-TATTCctCgAGAATTAAGTCATCGTC
oBB596	5′-TTTTGTAAgctAGCCATgataaataatc
oBB597	5′-atttatcATGGCTagcTTACAAAAAACAAG

##### Crystallization

Form A crystals of unliganded CodY were grown in hanging drops formed by mixing 1 μl of 10–20 mg·ml^−1^ CodY with 1 μl of well solution containing 1 m sodium citrate, pH 6.0, and 5% glycerol. Form B crystals were similarly prepared by mixing 1 μl of 10–20 mg·ml^−1^ CodY containing 10 mm GTP with 1 μl of well solution containing 0.1 m sodium citrate, pH 5.6, 1.7 m lithium sulfate, and 3% dioxane. Finally, form C crystals were prepared by mixing 1 μl of 10–20 mg·ml^−1^ CodY containing 5 mm GTPγS and 2 mm Tris(2-carboxyethyl)phosphine with 1 μl of well solution containing 0.1 m sodium citrate, pH 5.6, 20 mm Tris-HCl, pH 7.5, 35% ammonium sulfate. Crystals of CodY(L3S) in complex with isoleucine were grown in hanging drops prepared by mixing equal volumes of 18 mg·ml^−1^ protein containing 20 mm isoleucine with a well solution that contained 15% polyethylene glycol 5000, 5% tacsimate in 0.1 m HEPES buffer, pH 7.5.

##### Data Collection, Structure Solution, and Refinement

After transfer to solutions of mother liquor containing a suitable cryoprotectant, a preliminary X-ray analysis of crystals of CodY was carried out in-house using K_α_ radiation from a copper-rotating anode. Diffraction was initially very weak, but the quality of the diffraction data was significantly improved following cycles of crystal reannealing, *i.e.* by repeated thawing in cryoprotectant and cryo-cooling of the crystals. Full three-dimensional data were collected from the strongest diffracting crystals on beamlines at the Diamond Light Source (Harwell, UK) and the European Synchrotron Radiation Source (Grenoble, France) as detailed in Table 1. The data were processed and scaled in HKL2000 (52). As shown in Table 1, three crystal forms (A, B, and C) of unliganded CodY were obtained with 14, 10, and 2 molecules in the asymmetric unit, respectively.

The structure of the form B unliganded CodY crystals was solved by molecular replacement initially using the structure of the unliganded GAF (coordinate set 2GX5) domain as the search model. The initial molecular replacement solution of the latter had required pseudo-origin correction and gave an incentive to the development of the program Zanuda (53). Calculations in the program MOLREP (54) did not yield clear solutions because of the large number of molecules in the asymmetric unit. The resulting models were therefore examined manually and prioritized on the basis of whether they gave rise to GAF domain dimers reminiscent of that contained in the crystal structure of the search model. Suitable solutions were identified and fixed, and a subsequent search was carried out using the wHTH domain (coordinate set 1B0L).

The resulting structure was refined using REFMAC (55) with non-crystallographic symmetry restraints applied. This revealed electron density for the “missing” residues that form connecting helices. These were built manually in Coot (56). From the refined coordinates of this form B crystal structure, a dimer was used in molecular replacement calculations to solve the form A and form C crystal structures.

Later, the structure of CodY(L3S)-Ile was solved similarly by using coordinate set 2B18 representing the GAF domain with bound isoleucine followed by refinement using REFMAC (55). Figures of the structures of CodY were made using CCP4MG (57). The coordinates and structure factors for the models reported in this work have been deposited in the protein data bank with PDB codes 5LOO, 5LNH, and 5LOJ for the unliganded CodY crystal forms with 16, 10, and 2 molecules per asymmetric unit, respectively, and 5LOE for CodY(L3S) in complex with isoleucine.

##### Annealing DNA Oligonucleotides

Oligonucleotides, purified by HPLC, were purchased from Eurofins MWG Operon. Complementary oligonucleotides were resuspended in deionized water and incubated at a 1:1 molar ratio at a concentration of 50 μm in a buffer composed of 25 mm Tris, pH 8.5, 200 mm NaCl, 1 mm DTT, and 0.5 mm EDTA. The oligonucleotides were heated to 90 °C for 5 min to ensure full denaturation, followed by slow cooling to room temperature.

##### Labeling of DNA Fragments

The BcaPp8(19), BcaPp8(31), and BcaPp18(31) DNA duplexes were generated by annealing corresponding pairs of oligonucleotides (Table 3); the oligonucleotide mixtures in 10 mm Tris-Cl (7.5), 50 mm KCl were heated for 5 min in a boiling water bath and allowed to cool to room temperature. The BcaPp8(36) DNA duplex was synthesized by PCR using oligonucleotides oBB466 and oBB460 as primers and pBB1622 as template. In each case, one of the oligonucleotides was pre-labeled using T4 polynucleotide kinase and [γ-^32^P]ATP.

##### Gel Shift Experiments

Incubation of CodY with the ^32^P-labeled DNA duplexes was performed in 20 mm Tris-Cl, pH 8.0, 50 mm KCl, 2 mm MgCl_2_, 5% glycerol, 0.5 mm EDTA, 1 mm DTT, 0.05% Nonidet P, 40–25 μg/ml salmon sperm DNA as a binding buffer. Samples (10 μl) containing varying amounts of CodY and less than 1 fmol of DNA were incubated for 16 min at room temperature and separated on 8% non-denaturing 50 mm Tris, 384 mm glycine, 1 mm EDTA polyacrylamide gels in 35 mm HEPES, 43 mm imidazole buffer. In some experiments, 10 mm ILV was added to the incubation mixture, gel, and electrophoresis buffer. The gels were dried, and the radioactive bands were detected and quantified using storage screens, PhosphorImager, and the ImageQuant software (GE Healthcare).

##### SEC-MALLS

A Wyatt Dawn HELEOS-II 18-angle light scattering detector and Wyatt Optilab rEX refractive index monitor linked to a Shimadzu HPLC system and SPD20A UV-visible detector was used for SEC-MALLS. A Superdex S200 HR 10/30 size exclusion column was attached to the HPLC and equilibrated in a running buffer consisting of 50 mm Tris-HCl, pH 7.5, 150 mm NaCl in the presence or absence of 10 mm isoleucine. An SIL-20A Autosampler was used to inject 100-μl samples of 1 mg·ml^−1^ CodY samples, 19 μm DNA, or mixtures of the two. Data were analyzed with Astra software using a d*n*/d*c* value of 0.186 for protein and protein-DNA complexes and 0.168 for DNA (58, 59).

##### Introduction of the codY(L3S) Mutation in the B. subtilis Chromosome

A PCR fragment containing the full-length *codY* (L3S) gene and the flanking sequences was synthesized by two-step overlapping PCR. In the first step, the PCR product containing the 5′-part of the *codY* gene was synthesized by using oligonucleotide oBB594 as the forward primer and mutagenic oligonucleotide oBB596 as the reverse primer. In a similar manner, the PCR product containing the 3′-part of the *codY* gene was synthesized by using mutagenic oligonucleotide oBB597 as the forward primer and oligonucleotides oBB595 as the reverse primer. The PCR products were used in a second splicing step of PCR mutagenesis as overlapping templates to generate modified fragments containing the entire *codY* region; oligonucleotides oBB594 and oBB595 served as the forward and reverse PCR primers, respectively.

The spliced PCR product was digested with XbaI and XhoI and cloned in an integrative plasmid pBB1579 (*bgaB neo*) (60), creating pBB1756) (*codY*(L3S) *bgaB neo*). The latter plasmid was introduced by a single crossover homologous recombination event into the *bcaP* chromosomal locus of strain SMY. White Neo^s^ colonies indicating excision of pBB1756 from the chromosome were searched for on plates containing X-Gal (5-bromo-4-chloro-3-indolyl-β-d-galactopyranoside), the colored substrate of *bgaB*-encoded β-galactosidase. Multiple colonies were tested for acquisition of the *codY*(L3S) mutation by sequencing PCR products from the chromosomal *codY* allele.

##### Enzyme Assays

β-Galactosidase activity was determined as described previously (61).

## Author Contributions

A. L. S. and A. J. W. conceived the study and coordinated the work. V. M. L. determined the crystal structures with expert input from A. L. E. B, and V. M. L. made constructs for expression of purified proteins with E. B. then growing the crystals and V. L. Y. performing the biophysical experiments. B. R. B. performed the EMSA experiments and constructed and analyzed strains for *in vivo* analyses. The paper was written by A. J. W. with input from all authors.

## Footnotes

2 The abbreviations used are:

BCAA: branched-chain amino acid
wHTH: winged helix-turn-helix
SEC-MALLS: size exclusion chromatography with multiangle laser light scattering
GTPγS: guanosine 5′-3-*O*-(thio)triphosphate
r.m.s.Δ: root mean squared deviation.
